# Prognostic Significance of the Pluripotency Factors NANOG, SOX2, and OCT4 in Head and Neck Squamous Cell Carcinomas

**DOI:** 10.3390/cancers12071794

**Published:** 2020-07-04

**Authors:** Daniel Pedregal-Mallo, Francisco Hermida-Prado, Rocío Granda-Díaz, Irene Montoro-Jiménez, Eva Allonca, Esperanza Pozo-Agundo, Mónica Álvarez-Fernández, César Álvarez-Marcos, Juana M. García-Pedrero, Juan Pablo Rodrigo

**Affiliations:** 1 Department of Otolaryngology, Hospital Universitario Central de Asturias, 33011 Oviedo, Spain; pedregal.dm@gmail.com (D.P.-M.); caalvarez@uniovi.es (C.Á.-M.); 2 Department of Head and Neck Cancer, Instituto de Investigación Sanitaria del Principado de Asturias (ISPA), 33011 Oviedo, Spain; franjhermida@gmail.com (F.H.-P.); rocigd281@gmail.com (R.G.-D.); imj21897@gmail.com (I.M.-J.); ynkc1@hotmail.com (E.A.); espe6196@hotmail.com (E.P.-A.); maf.finba@gmail.com (M.Á.-F.); 3 Instituto Universitario de Oncología del Principado de Asturias (IUOPA), Universidad de Oviedo, 33006 Oviedo, Spain; 4 Ciber de Cáncer (CIBERONC), Instituto de Salud Carlos III, 28029 Madrid, Spain

**Keywords:** head and neck squamous cell carcinoma, NANOG, SOX2, OCT4, prognosis, lymph node metastasis

## Abstract

Cancer stem cells (CSCs) play major roles in tumor initiation, progression, and resistance to cancer therapy. Several CSC markers have been studied in head and neck squamous cell carcinomas (HNSCC), including the pluripotency factors NANOG, SOX2, and OCT4; however, their clinical significance is still unclear. NANOG, SOX2, and OCT4 expression was evaluated by immunochemistry in 348 surgically-treated HNSCC, and correlated with clinicopathological parameters and patient outcomes. mRNA expression was further analyzed in 530 *The Cancer Genome Atlas* (TCGA) HNSCC. NANOG protein expression was detected in 250 (72%) cases, more frequently in patients with lymph node metastasis (*p* = 0.003), and was an independent predictor of better survival in multivariate analysis. While OCT4 expression was undetectable, SOX2 expression was observed in 105 (30%) cases, and strongly correlated with NANOG expression. Combined expression of both proteins showed the highest survival rates, and double-negative cases the worst survival. Strikingly, the impact of NANOG and SOX2 on outcome varied depending on tumor site and lymph node infiltration, specifically showing prognostic significance in pharyngeal tumors. Correlation between NANOG and SOX2 at mRNA and protein was specifically observed in node positive (N+) patients, and consistently correlated with better survival rates. According to our findings, NANOG protein expression is frequent in HNSCC, thereby emerging as an independent predictor of better prognosis in pharyngeal tumors. Moreover, this study uncovers a differential impact of NANOG and SOX2 expression on HNSCC prognosis, depending on tumor site and lymph node infiltration, which could facilitate high-risk patient stratification.

## 1. Introduction

Head and neck cancer is the seventh most common cancer worldwide, with an incidence of more than 890,000 new cases annually, and a mortality of 450,000 deaths each year [1]. More than 90% are head and neck squamous cell carcinomas (HNSCC), arising in the mucosal surfaces of the upper aerodigestive tract. The main risk factors for the development of HNSCC include tobacco smoking and excessive alcohol consumption, which show a synergistic effect [2]. Other factors, such as genetic background, viral infection by human papillomavirus (HPV), diet, geographic, and socioeconomic factors have also been related to HNSCC. Despite the continuous advances in treatment options, the survival of HNSCC patients has barely improved [1,2]. One of the major reasons and challenges for clinical management is the development of treatment resistance that ultimately leads to disease control failure. This has been attributed to the persistence of highly resistant cancer stem cell (CSC) subpopulations.

Many evidences indicate that tumors are complex heterogeneous structures with very distinct cell subpopulations composing the tumor mass, including a subset of pluripotent cells known as CSCs. These cells exhibit properties usually associated with embryonic stem cells, and it has been demonstrated that CSCs contribute to tumorigenesis, tumor differentiation, maintenance, spread, and recurrence [3]. Consequently, the presence of CSC subpopulations with self-renewal capacity and multipotency properties could also have an important impact on the prognosis of HNSCC patients.

The pluripotency factors NANOG, SOX2, and OCT4 have been reported as CSC markers and key regulators in HNSCC and other cancers [4,5,6,7,8,9,10,11,12]. These transcription factors are able to maintain the self-renewal capacity in embryonic stem cells. Their combined overexpression has been used to identify CSC populations in squamous cell carcinomas [13,14,15]. OCT4 plays an important role in maintaining pluripotency and undifferentiated status in embryonic stem cells [16] and has been associated with tumor progression and poor prognosis in HNSCC [14,17]. NANOG is a downstream target of OCT3/4 [18], and, similarly to that factor, has been correlated with tumorigenesis, loss of differentiation, invasion, and metastasis [14,17]. SOX2 is a transcription factor belonging to the SOX family, essential for self-renewal in stem cells and neural progenitor cells [19]. SOX2 takes part in cancer stemness and its expression has been associated with CD44 and ALDH1 expression and correlated with oral squamous cell carcinoma (OSCC) metastasis [20].

Patients who suffer from HNSCC experiment with very different clinical outcomes in spite of standardized treatments, so a better knowledge of molecular profiles and their impact on patients’ prognosis is needed. To this purpose, the present study investigates the role of pluripotency factors NANOG, SOX2, and OCT4 in a large homogeneous cohort of 348 HNSCC patients to ascertain their potential impact on patient prognosis and disease outcome.

## 2. Patients and Methods

### 2.1. Patients and Tissue Specimens

We retrospectively collected surgical tissue specimens from HNSCC patients who underwent resection of their tumors at the Hospital Universitario Central de Asturias between 1990 and 2010, in accordance with approved institutional review board guidelines. All experimental procedures were conducted in accordance with the Declaration of Helsinki and approved by the Institutional Ethics Committee of the Hospital Universitario Central de Asturias and by the Regional CEIC from Principado de Asturias (date of approval 5 May 2016; approval number: 70/16) for the PI16/00280 project. The formalin-fixed, paraffin-embedded tissue samples and data from donors included in this study were provided by the Principado de Asturias BioBank (PT17/0015/0023), integrated in the Spanish National Biobanks Network, and they were processed following standard operating procedures with the appropriate approval of the Ethical and Scientific Committees. A large unbiased homogenous cohort of surgically-treated HNSCC patients was selected for study according to the following criteria: (a) having a single primary surgically-treated tumor in the oropharynx, hypopharynx, or larynx; (b) confirmed microscopically clear surgical margins; (c) no treatments prior to surgery; (d) HPV-negative tumors; (e) a minimum follow up of five years.

Information on HPV status was available for all the patients. HPV detection was performed using p16 immunohistochemistry, high-risk HPV DNA detection by in situ hybridization, and genotyping by GP5+/6+-PCR, as previously reported [21,22].

For mRNA analysis, surgical tissue specimens from 15 patients with HNSCC who underwent surgical treatment at the Hospital Universitario Central de Asturias were prospectively collected, following institutional review board guidelines. Patient-matched normal mucosa was also collected. Biopsies were sharply excised, placed in sterile tubes, and stored in RNA later at −80 °C. Primary keratinocytes from non-oncologic patients without exposure to tobacco carcinogens were used as controls.

### 2.2. Tissue Microarray (TMA) Construction and Immunohistochemistry

Three morphologically representative areas (1 mm cylinders) were selected from each individual tumor paraffin block for the construction of tissue microarrays (TMAs) as described previously [21], containing a total of 348 HNSCC (229 oropharyngeal, 60 hypopharyngeal, and 59 laryngeal carcinomas). In addition, each TMA included three cores of normal epithelium (tonsillar, pharyngeal, and laryngeal mucosa obtained from non-oncologic patients) as an internal negative control.

The TMAs were cut into 3 μm sections and dried on Flex IHC microscope slides (Dako, Glostrup, Denmark). The sections were deparaffinized with standard xylene and hydrated through graded alcohols into water. Antigen retrieval was performed using Envision Flex Target Retrieval solution, high pH (Dako). Staining was done at room temperature on an automatic staining workstation (Dako Autostainer Plus, Dako, Glostrup, Denmark) using NANOG (D73G4) XP^®^ rabbit monoclonal antibody (Cell Signaling Technology, Inc. #4903S, Leiden, The Netherlands) at 1:200 dilution, anti-SOX2 rabbit polyclonal antibody (Merck Millipore #AB5603) at 1:1000 dilution, and anti-OCT4 antibody (Abcam, Cambridge, UK #ab19857) at 1:2000 dilution. Immunodetection was carried out with the Dako EnVision Flex + Visualization System (Dako Autostainer, Dako, Glostrup, Denmark), using diaminobenzidine as chromogen. Counterstaining with hematoxylin was the final step.

A sample of testicular seminoma (a tumor known to express OCT4, NANOG, and SOX2) was used as positive control.

For NANOG expression, a semiquantitative scoring system based on staining intensity was applied, as previously established [23], and divided into three categories: negative (absence of staining, score 0), weak to moderate (some cytoplasmic staining in tumor areas, score (1)), and strong protein expression (intense and homogeneous cytoplasmic staining in tumor areas, score (2)), with an interobserver concordance higher than 95%. For statistical purposes, NANOG staining was dichotomized as negative (score 0) versus positive (scores 1–2). SOX2 and OCT4 staining was evaluated as the percentage of tumor cells with nuclear staining. SOX2 staining scores were classified as negative or positive expression on the basis of values below or above the cut-off value of 10%.

### 2.3. RNA Extraction and Real-Time RT-PCR

Total RNA was extracted from fresh-frozen tissue samples and normal keratinocytes using TRIzol reagent (Invitrogen Life Technologies, Carlsbad, CA, USA), and cDNA was synthesized with the Superscript II RT-PCR System (Invitrogen Life Technologies, Carlsbad, CA, USA), according to manufacturer’s protocols.

Gene expression was analyzed by real-time PCR using SYBR Green Master Mix protocol (Applied Biosystems, Foster City, CA, USA) in a StepOnePlus Real-Time PCR System (Applied Biosystems, Foster City, CA, USA). Reactions were run in triplicates using the specific primers detailed in Table S2, and the ribosomal coding gene RPL19 was used as endogenous control. Relative mRNA expression was calculated using the 2^−ΔΔCT^ method and the data were expressed as the fold change in NANOG1 or SOX2 levels in the tumor sample (or matched normal epithelium) normalized to L19 levels and relative to the primary keratinocytes used as controls.

### 2.4. Statistical Analysis

χ^2^ and Fisher’s exact tests were used for comparison between categorical variables. For time-to-event analysis, Kaplan–Meier curves were plotted. Differences between survival times were analyzed by the log-rank method. Cox proportional hazards models were utilized for univariate and multivariate analyses. The hazard ratios (HR) with 95% confidence interval (CI) and *p* values were reported. All tests were two-sided. *p* values of ≤0.05 were considered statistically significant.

## 3. Results

### 3.1. Patient Characteristics

Three hundred forty-eight HNSCC patients were enrolled in this study, following the above-described inclusion criteria. Only 12 patients were women, and the mean age was 59 years (range 36 to 86 years). Most patients were habitual tobacco smokers, 196 moderate (1–50 pack-years) and 147 heavy (>50 pack-years); 321 patients were alcohol drinkers. The distribution by location was 229 oropharyngeal, 60 hypopharyngeal, and 59 laryngeal tumors. The tumors were classified according to the TNM classification system (7th edition, International Union Against Cancer): 17 tumors were classified as stage I, 21 stage II, 59 stage III, and 251 stage IV. The series included 135 well-, 139 moderately and 73 poorly differentiated tumors, determined according to the degree of differentiation of the tumor (Broder’s classification). Two hundred sixteen (62%) of 348 patients received postoperative radiotherapy. The main clinicopathological features by site are shown in Table S1.

### 3.2. NANOG, SOX2, and OCT4 Protein Expression in HNSCC Tissue Specimens

**Figure 1:**
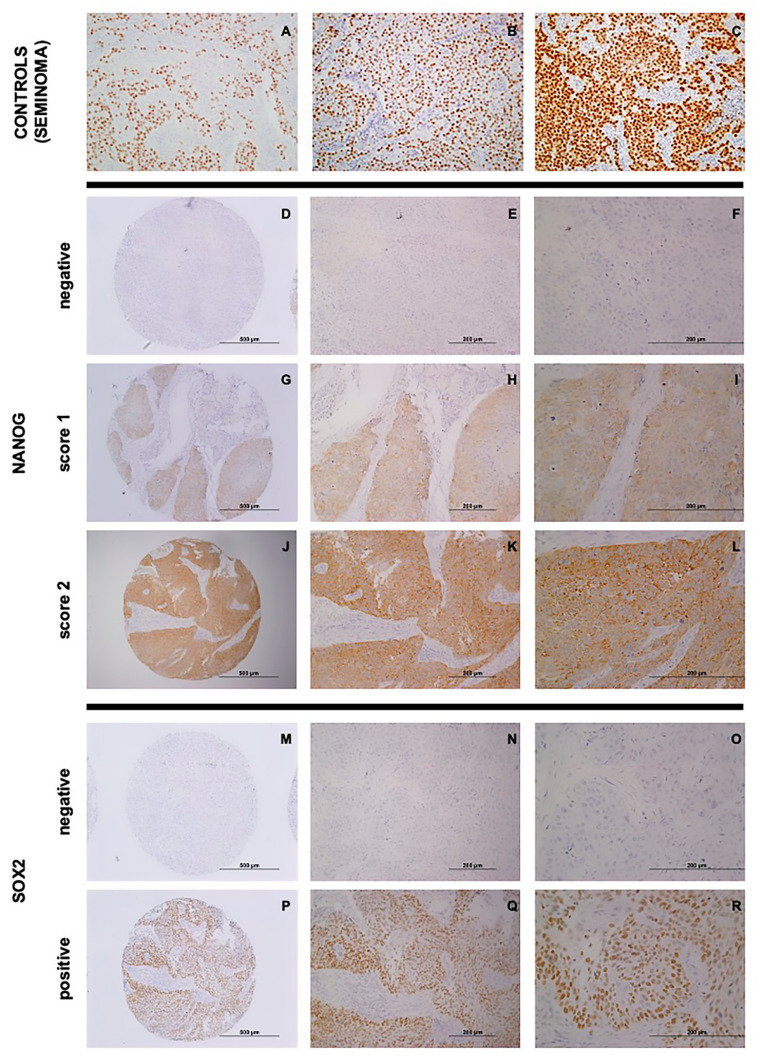
Immunohistochemical analysis of NANOG, SOX2, and OCT4 expression in head and neck squamous cell carcinoma (HNSCC). (**A**–**C**) Positive controls of human seminoma samples for NANOG (**A**), SOX2 (**B**), and OCT4 (**C**) expression. Representative examples of HNSCC showing negative NANOG staining, score 0 (**D** 100×, **E** 200×, **F** 400×); cytoplasmic NANOG staining, score 1 (**G** 100×, **H** 200×, **I** 400×); and cytoplasmic NANOG staining, score 2 (**J** 100×, **K** 200×, **L** 400×). HNSCC samples showing negative nuclear SOX2 staining (**M** 100×, **N** 200×, **O** 400×), and positive nuclear SOX2 staining (**P** 100× **Q** 200×, **R** 400×).

Strong nuclear staining was detected in human seminoma, which was used as a positive control for these three proteins (Figure 1A–C). Two hundred fifty (72%) out of 348 tumors exhibited positive NANOG expression (scores 1–2) (Figure 1D–L), showing a predominantly cytoplasmic pattern, but also some nuclear staining, whereas NANOG expression was negligible in both normal epithelium and stromal cells. In addition, positive nuclear SOX2 expression in >10% of tumor cells was detected in 105 (30%) tumor samples (Figure 1M–R). None of the tumor samples showed OCT4 expression.

There was a strong positive correlation between NANOG and SOX2 expression: 96 (91%) of the 105 SOX2-positive cases showed positive NANOG expression (Spearman coefficient 0.286, *p* < 0.001).

### 3.3. Associations with Clinicopathological Parameters

The relationships between NANOG and SOX2 expression and the clinicopathological parameters are shown in Table 1. Positive NANOG expression was significantly associated with node positive (N+) tumors (*p* = 0.003) and hypopharyngeal tumors (*p* = 0.019), and was also more frequent in poorly differentiated (*p* = 0.084) and advanced stage (*p* = 0.204) tumors. In contrast, positive SOX2 expression was significantly more frequent in laryngeal tumors (*p* = 0.002). No other significant associations between SOX2 expression and clinical characteristics were observed.

### 3.4. Impact on Patient Prognosis

**Figure 2:**
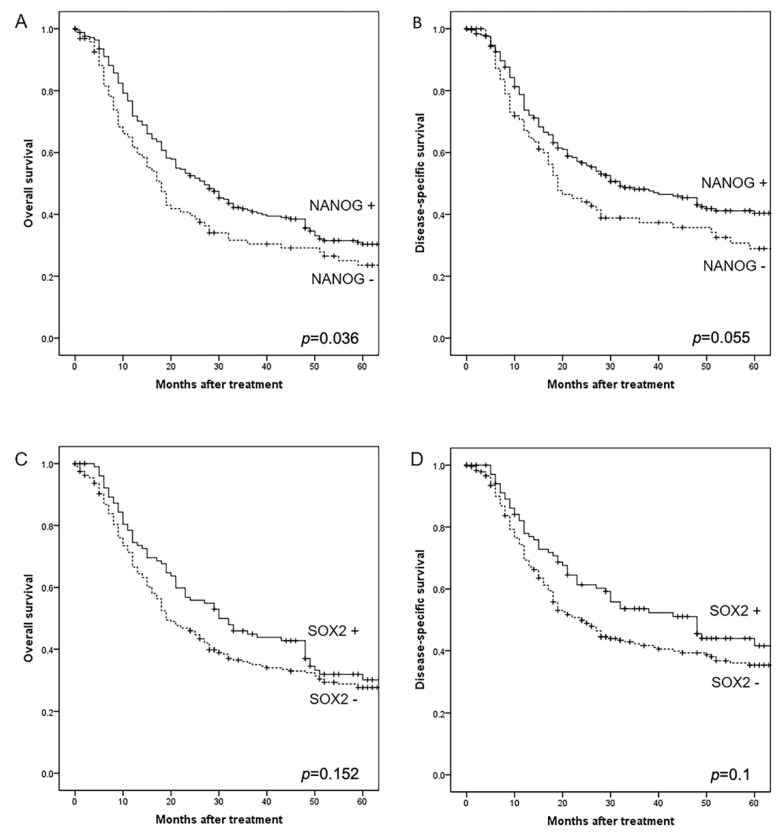
Kaplan–Meier overall and disease-specific survival curves in the whole series categorized by NANOG (**A**,**B**) and SOX2 expression (**C**,**D**).

Patients harboring positive NANOG expression exhibited significantly higher disease-specific survival (DSS) and overall survival (OS) rates (log-rank, *p* = 0.055 and *p* = 0.012, respectively; Figure 2A,B). Similarly, patients with positive SOX2 expression also showed higher DSS and OS rates; however, these differences did not reach statistical significance (Figure 2C,D).

On multivariate analysis, the variables with significant influence on DSS were T and N classification, histopathological grade, and NANOG expression (Table 2).

Given the relationship between NANOG and SOX2 expression and function, the impact of NANOG and SOX2 on patient prognosis was studied separately, as was the combination of both factors (Figure S1). Patients with NANOG and/or SOX2 expression had better survival rates than patients harboring double-negative expression (five-year DSS 41% vs. 26%; log-rank, *p* = 0.019). Among the subset of patients with SOX2-negative expression, those harboring NANOG-positive expression showed better overall survival rates (five-year OS 31% vs. 17%; log-rank, *p* = 0.011). On the other hand, patients with NANOG-negative expression did not show differences in survival rates based on SOX2 immunostaining (five-year OS 44% vs. 17%; log-rank, *p* = 0.266), not surprisingly, considering that only nine NANOG-negative patients had positive SOX2 expression (Figure S1).

We also found important differences in the prognostic significance of NANOG and SOX2 expression, depending on the tumor site. Table 3 summarizes the mean DSS and OS for patients categorized by individual and combined expression of NANOG and SOX2 in the total cohort of 348 HNSCC patients, or the subgroups of oropharyngeal tumors (*n* = 229), hypopharyngeal tumors (*n* = 60), and laryngeal tumors (*n* = 59). While either NANOG expression or double-positive expression of NANOG and SOX2 were significantly correlated with improved survival rates in patients harboring pharyngeal tumors, none of these factors showed any impact on the clinical outcome of patients with laryngeal tumors.

**Figure 3:**
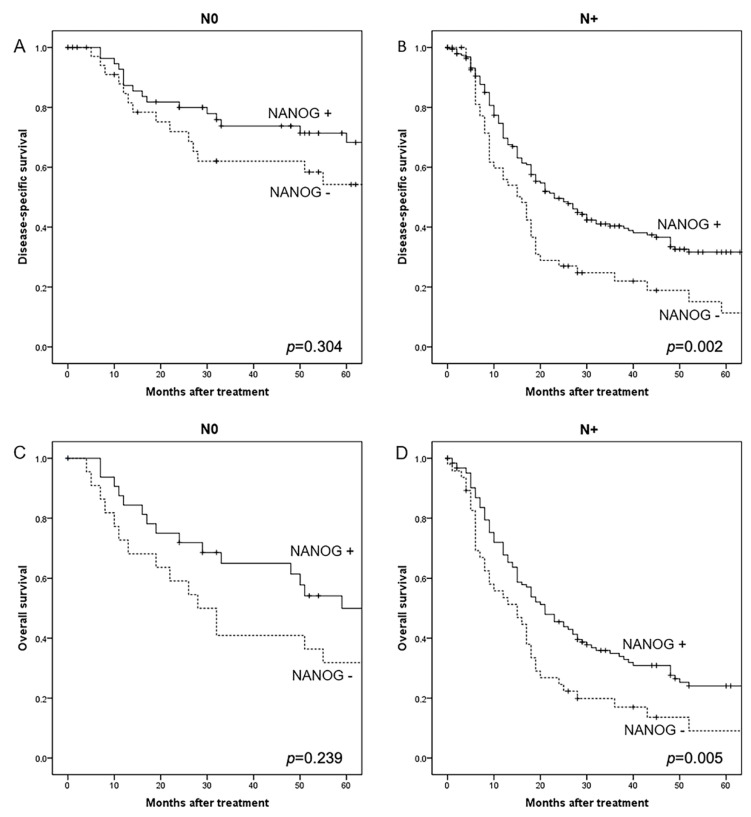
Kaplan–Meier disease-specific survival curves categorized by NANOG expression in N0 (**A**) and N+ patients (**B**), and overall survival curves categorized by NANOG expression in N0 (**C**) and N+ patients (**D**).

Noteworthy, the prognostic significance of NANOG expression was influenced by the presence of cervical lymph node metastasis (Figure 3). In N+ patients, NANOG expression was significantly correlated with a better disease-specific survival (five-year DSS was 32% in NANOG-positive vs. 11% in NANOG-negative cases; log-rank, *p* = 0.002), and also a better overall survival (log-rank, *p* = 0.005). In marked contrast, in the N0 subgroup of patients, NANOG expression was not associated with DSS (log-rank, *p* = 0.304) nor OS (log-rank, *p* = 0.239). Moreover, the association between NANOG and SOX2 expression was also dependent on pN classification. Thus, a strong correlation was specifically observed in N+ patients (Spearman coefficient 0.308, *p* < 0.001), but not in N0 patients (Spearman coefficient 0.029, *p* = 0.831).The impact of SOX2 expression on patient prognosis also varied with pN classification, although the differences in DSS and OS did not reach statistical significance (Figure S2).

### 3.5. In Silico Analysis of NANOG and SOX2 mRNA Expression Using The Cancer Genome Atlas Data

In an attempt to further confirm and validate our findings, the clinical relevance of NANOG and SOX2 mRNA expression was investigated by analyzing a cohort of 530 HNSCC patients from *The Cancer Genome Atlas* (TCGA) [24] using the platform cBioPortal (http://cbioportal.org/) [25]. Correlations with pN classification were assessed in a subset of 424 HNSCC patients with available data on lymph node stage. Patients were categorized by NANOG and SOX2 mRNA expression (high expression versus low expression) according to the pN classification. First, the correlation between NANOG and SOX2 mRNA expression was assessed separately in N0 (*n* = 177) and N+ patients (*n* = 246) (Figure S3). Concordantly with our protein data, a strong correlation between the mRNA levels of NANOG and SOX2 was specifically observed in N+ patients from the TCGA HNSCC cohort (Spearman coefficient 0.21, *p* = 0.001), but not in the N0 patients (Spearman coefficient 0.12, *p* = 0.104). Furthermore, NANOG and SOX2 mRNA expression differentially influenced patient prognosis depending on pN classification. Thus, high mRNA levels of NANOG and SOX2 were found to significantly associate with improved survival rates in N+ patients (log-rank, *p* = 0.032 and *p* = 0.036, respectively) (Figure S4), and more strongly observed in N2+ patients (log-rank, *p* = 0.009 and *p* = 0.004, respectively) (Figure S5). By contrast, in the N0 subgroup, patients with high NANOG mRNA levels experienced a worse, although not significant, survival than those with low NANOG expression (median OS 57.47 months vs. 89.32 months; log-rank, *p* = 0.139), whereas SOX2 mRNA levels had no major impact on outcome (median OS 88.86 months vs. 84.49 months; log-rank, *p* = 0.523) (Figure S4).

As an extension of these data, NANOG and SOX2 mRNA expression was also analyzed by real-time RT-PCR in a prospective series of 15 fresh-frozen HNSCC tissue specimens and patient-matched normal epithelia. NANOG and SOX2 levels were found to frequently and significantly increase in the tumors compared to patient-matched normal epithelia and primary keratinocytes (used as non-oncologic control without exposure to tobacco carcinogens) (Figure S6). Thus, NANOG mRNA levels in the tumors ranged between 0.23- and 65.59-fold (mean 12.08), whereas in the corresponding normal counterparts they ranged between 0.20- and 27.84-fold (mean 4.35) (*p* = 0.06; Student’s t-test). SOX2 mRNA levels were also higher in the tumors ranging between 61.87- and 636.4-fold (mean 276.6), whereas the matched normal epithelia ranged between 0.86- and 399.9-fold (mean 78.05) (*p* = 0.005; Student’s *t*-test). Interestingly, histologically normal mucosa from various patients (33.3%, 5/15 cases) also exhibited increased expression of NANOG and SOX2, showing comparable mRNA levels in both the tumor sample and the patient-matched normal tissue. These molecular alterations could be related to the cancerization field due to exposure of the entire epithelial mucosa to tobacco carcinogens.

## 4. Discussion

The role of CSCs in head and neck tumors has been widely investigated during the last years. Several CSC markers have been described in HNSCC, such as CD44, BMI-1, CD133, ALDH1, also including the pluripotency factors NANOG, OCT4, and SOX2 [6,7,26]. However, the roles of OCT4, NANOG, and SOX2 in HNSCC prognosis is still unclear, as varying results have been reported by several groups. This prompted us to jointly investigate the significance of NANOG, SOX2, and OCT4 expression in a large unbiased cohort of HNSCC patients, and their possible influence on the clinical outcome. To this purpose, a large homogeneous series of 348 surgically-treated HPV-negative HNSCC patients was selected for study.

We have recently demonstrated that expression of NANOG and SOX2 plays a relevant role in early stages of laryngeal tumorigenesis. The expression of both proteins was found to increase in patients with precancerous lesions and, more importantly, strongly predicted the risk of progression to invasive carcinoma [23,27]. In addition, both factors have been involved in the epithelial to mesenchymal transition. NANOG expression has been inversely correlated with E-cadherin expression and positively with high N-cadherin expression. High SOX2 expression has also been related with high expression of N-cadherin, but not E-cadherin expression [28]. Altogether, these results encourage us to investigate the role of NANOG and SOX2 expression in late stages of HNSCC progression and disease outcome.

Through a cooperative interaction, SOX2 and OCT4 drive pluripotent-specific expression of different genes. OCT4 and SOX2 form a heterodimer and bind to *NANOG* promoter in embryonic stem cells, which is crucial for NANOG gene transcription. In relation to this, a strong positive correlation between SOX2 and NANOG expression was found at both mRNA and protein levels using two large independent cohorts of HNSCC patients. Nevertheless, even though mRNA expression levels were similar (35% high NANOG and 43% high SOX2), positive NANOG protein expression was, however, detected at a much higher frequency (70%) than SOX2 expression (30%). In fact, 156 patients with positive NANOG expression concomitantly showed negative SOX2 expression in our cohort. Mechanistically, this finding reflects the partial contribution of SOX2 as a regulator of NANOG protein expression in HNSCC, and points to additional post-transcriptional mechanisms as further responsible for the high percentage of NANOG-positive cases observed. Similarly, differences in expression regulation and prognostic significance between NANOG and SOX2 have also been revealed in lung tumors, thus reinforcing its complexity and heterogeneity. In lung adenocarcinomas, expression of SOX2 but not NANOG was detected in approximately 50% of stage I tumors [29], whereas another study reported NANOG expression without SOX2 [30], hence suggesting a SOX2-independent regulatory mechanism of NANOG expression in these tumors. In lung squamous cell carcinoma, a good correlation between NANOG and SOX2 expression was observed; however, there was no clear correlation with survival outcome [30]. Contrasting to this, NANOG has been associated with good prognosis [31], and SOX2 was found to be an independent factor of good prognosis [32]. Notably, the mRNA expression levels of both NANOG and SOX2 were found to be downregulated in subpopulations of cancer stem cells isolated from lung cancer tissue specimens [33].

On the other hand, the analysis of NANOG and SOX2 mRNA expression in a prospective series of HNSCC samples and patient-matched normal epithelia further revealed that NANOG and SOX2 levels were consistently found to increase in tumors compared to the matched normal mucosa. However, strikingly, a subset of HNSCC patients (33.3%) harbored high expression of NANOG and SOX2 in histologically normal mucosa with comparable levels to the matched tumor. This finding reflects the early occurrence of altered NANOG and SOX2 expression in head and neck carcinogenesis, also frequently detected in precancerous lesions [23,27,34], plausibly triggered by continuous exposure of the epithelial mucosa to tobacco-related carcinogens. In line with this, it has been demonstrated that nicotine plays a critical role in the development of tobacco-induced cancers by increasing the expression of various CSC markers NANOG, OCT4, CD44, and BMI-1 and regulating CSC properties and tumorigenic potential in HNSCC models in vitro and in vivo [35]. It has also been recently reported that NANOG expression significantly correlates with smoking and alcohol drinking habits in OSCC patients [34]. Similarly, nicotine and e-cigarette extracts have been found to regulate SOX2 expression and stemness through a nicotinic acetylcholine receptor (nAChR)-YAP1-E2F1 signaling axis [36]. Together, these data uncover a relationship between stemness by means of important CSC regulators, such as NANOG and SOX2, and classical chemical carcinogens in HNSCC and other tobacco-related cancers.

NANOG has been found overexpressed in different cancer types, including OSCC [4] and laryngeal squamous cell carcinoma (LSCC) [23]. Functionally, NANOG overexpression has been implicated in tumor transformation, tumorigenicity, metastasis, and is also correlated with poor differentiation status and chemoresistance [37]. Moreover, NANOG expression has also been associated with a better or worse prognosis in different cancers, including OSCC [34,38]. In a meta-analysis about the prognostic significance of NANOG that included nine studies on HNSCC, NANOG expression was associated with lower survival rates [39]. Contrasting with this, NANOG expression was associated with a better prognosis in our cohort and was an independent predictor in multivariate analysis. Our study also uncovered striking differences in the prognostic impact of NANOG expression, depending on the tumor site and lymph node infiltration. Thus, NANOG expression, and to a lesser extent SOX2, specifically predicted good prognosis in patients with pharyngeal but not laryngeal tumors. In addition, the impact of NANOG expression was greater in patients with lymph node metastasis than in node-negative patients, as was consistently demonstrated at both mRNA and protein levels using two large independent HNSCC cohorts. Therefore, since the clinical relevance of NANOG expression is influenced by some clinicopathological characteristics, differences in tumor localization and N classification of the patients included could explain the discrepancies between reported studies.

SOX2 expression has also been implicated in different biological processes that regulate tumor progression, such as cell proliferation, migration, invasion, tumorigenesis, antiapoptosis, and chemoresistance [40,41]. Despite multiple studies published so far exploring the role of SOX2 in cancer, its impact on disease outcome remains controversial. High expression of SOX2 has been associated with a poor prognosis in some studies [8,9,42]. However, other groups have reported that high expression of SOX2 is associated with absence of lymph node metastasis and a better prognosis in patients with OSCC [43], and also with improved clinical outcome in HNSCC patients treated with chemoradiotherapy [44]. Similarly, Chung et al. and Bochen et al. found a positive association between high SOX2 expression and HNSCC patient prognosis [45,46]. In our series, SOX2 expression was associated with a better DSS, although not significant. A possible pleiotropic function of SOX2 in squamous cell carcinomas from other locations has been suggested, based on differential interactions with other important factors that could cooperatively contribute to the development and progression of these tumors [47]. The binding to different cofactors and enhancement or repression of other factors could result in opposed clinical outcomes, thereby explaining the apparently contradictory results.

Given the strong relationship between NANOG and SOX2 expression and function, as a further step, we analyzed the combined effect of NANOG and SOX2 expression as an integrated risk factor. We observed that patients with positive expression of both proteins exhibited the best prognosis, while the subgroup of double-negative patients clearly showed the worst prognosis. The integrated risk score could be assessed from preoperative biopsies and serve as an additional prognostic parameter. Together with other clinical parameters such as resection margins and extracapsular extension, it could help to improve patient stratification and the identification of high-risk subgroups for treatment intensification.

OCT4 has also been related to various oncogenic processes [5], such as tumor transformation, tumorigenicity, invasion, and metastasis in OSCC [15]. OCT4 expression has been widely used to identify CSC subpopulations in several carcinomas, in conjunction with other CSC markers [20,48,49]. High expression of OCT4 and NANOG has been associated with a lower OS in HNSCC patients [50]. Other publications have found a worse prognosis in patients with high expression of both NANOG and OCT4 than those with high expression of NANOG or OCT4 alone [4]. However, OCT4 expression was not detected in any of the HNSCC samples in our cohort despite the fact that strong positive nuclear staining was indeed observed in a seminoma sample used as a positive control. These varying findings could be the result of different antibodies employed for immunostaining, targeting different epitopes or different specificity for OCT4 protein isoforms. OCT4A is responsible for the pluripotency properties of embryonic stem cells, but OCT4B cannot sustain these properties [51]. Different properties and/or OCT4 isoforms could be the main cause of confusion and controversies on the role of OCT4 in the different cancer types. Noteworthy, the same OCT4 antibody herein used has been proven to successfully detect OCT4 expression in sarcoma samples by immunohistochemistry, and although OCT4 expression was strongly correlated with SOX2 expression, unlike the former, the latter was the most prevalent, and was the only one found to be clinically relevant in these tumors [52].

## 5. Conclusions

Our results show that NANOG protein expression is frequent in HNSCC and emerges as an independent predictor of better clinical outcome, specifically in pharyngeal but not laryngeal tumors. SOX2 expression, although less frequent, was strongly correlated with NANOG expression. Combined expression of both proteins had a stronger prognostic significance, probably suggesting a cooperative functional role between both proteins. Striking differences were uncovered regarding the clinical impact of NANOG and SOX2 expression on patient outcome, with distinct prognostic relevance depending on tumor site and lymph node infiltration. These novel findings could facilitate patient management and high-risk stratification, and also provide a plausible explanation to reconcile contradictory published data about the prognostic significance of these factors.

## Figures and Tables

**Table 1:** Correlations between NANOG and SOX2 expression and clinicopathological characteristics.

Characteristic	No.	NANOG-Positive Expression (%)	*p* Value	SOX2-Positive Expression (%)	*p* Value
**pT classification**					
T1–T2	106	80 (75)	0.350	36 (34)	0.163
T3	116	78 (67)		39 (34)	
T4	125	92 (74)		30 (24)	
**pN classification**					
N0	94	56 (60)	0.003	29 (31)	0.896
N1–3	254	194 (76)		76 (30)	
**Disease stage**					
I–II	38	25 (66)	0.204	12 (32)	0.754
III	59	38 (64)		20 (34)	
IV	251	187 (75)		73 (29)	
**Pathological grade**					
Well-differentiated	135	88 (65)	0.084	40 (30)	0.740
Moderately differentiated	139	104 (75)		45 (32)	
Poorly differentiated	73	57 (78)		20 (27)	
**Site**					
Oropharynx	229	157 (69)	0.019	56 (24)	0.002
Hypopharynx	60	52 (87)		21 (35)	
Larynx	59	41 (69)		28 (47)	
**Total cases**	**348**	**250 (72)**		**105 (30)**	

**Table 2:** Multivariate survival analysis (Cox regression).

Variables	HR	95% CI	*p*
T (3–4/1–2)	1.153	1.033–1.288	0.011
N (+/−)	2.619	1.756–3.906	0.000
G (3/1–2)	1.494	1.071–2.085	0.018
Location (pharynx/larynx)	0.695	0.448–1.077	0.103
NANOG (+/−)	0.783	0.662–0.927	0.004
SOX2 (+/−)	0.961	0.685–1.349	0.820

**Table 3:** Correlation between NANOG and SOX2 expression, and 5-year survival rates.

Patients	NANOG-Positive Expression	NANOG-Negative Expression	*p*	SOX2-Positive Expression	SOX2-Negative Expression	*p*	Double-Positive Expression	Double-Negative Expression	*p*
**Whole tumor series**									
DSS	40%	29%	0.055	42%	35%	0.101	40%	26%	0.019
OS	28%	20%	0.012	26%	26%	0.221	24%	17%	0.015
**Oropharyngeal tumors**									
DSS	43%	22%	0.013	43%	35%	0.312	40%	18%	0.031
OS	29%	16%	0.011	28%	24%	0.319	25%	13%	0.027
**Hypopharyngeal tumors**									
DSS	26%	14%	0.126	20%	26%	0.420	20%	14%	0.095
OS	21%	13%	0.049	14%	23%	0.696	14%	13%	0.106
**Laryngeal tumors**									
DSS	52%	58%	0.580	57%	50%	0.681	57%	59%	0.734
OS	36%	38%	0.951	34%	38%	0.885	32%	36%	0.887

## Supplementary Materials

The following are available online at https://www.mdpi.com/2072-6694/12/7/1794/s1, Table S1: Clinicopathological characteristics of the studied cohort of 348 HNSCC patients, Table S2: Primers used for real-time RT-PCR, Figure S1: Kaplan–Meier survival curves based on independent and combined expression of NANOG and SOX2, Figure S2: Kaplan–Meier overall survival curves categorized by SOX2 expression in the subgroups N0, N+, and N2+, Figure S3: Correlation between NANOG and SOX2 mRNA expression in a cohort of 530 HNSCC patients from *The Cancer Genome Atlas* (TCGA), Figure S4: Prognostic implications of NANOG and SOX2 mRNA expression in a subset of 424 HNSCC patients with lymph node stage data from the TCGA HNSCC cohort, Figure S5: Prognostic implications of NANOG and SOX2 mRNA expression in the subset of 179 N2+ patients from the TCGA HNSCC cohort, Figure S6: Analysis of NANOG and SOX2 mRNA levels by real-time RT-PCR in 15 fresh HNSCC tumor samples and patient-matched normal epithelia.
