# Supplementary material for: Prognostic Significance of the Pluripotency Factors NANOG, SOX2, and OCT4 in Head and Neck Squamous Cell Carcinomas
Source: Cancers (Basel). 2020 Jul 4;12(7):1794. doi: 10.3390/cancers12071794 (PMC7408284; doi:10.3390/cancers12071794)
Supplement: Supplementary file 1 [file cancers-12-01794-s001.pdf]

SUPPLEMENTARY INFORMATION

Supplementary Table S1. Clinicopathological characteristics of the studied cohort of 348 HNSCC patients.

	Oropharynx (%)	Hypopharynx (%)	Larynx (%)
**Patients**	229 (66)	60 (17)	59 (17)
**Gender**			
Male	221 (64)	57 (16)	58 (17)
Female	8 (2)	3 (1)	1 (0.3)
**Age**			
Average	58	59	62
Range	36 – 85	43 – 80	36 – 86
**pT classification**			
T1-T2	70 (20)	18 (5)	18 (5)
T3-T4	159 (46)	42 (12)	41 (12)
**pN classification**			
N0	56 (16)	9 (2)	29 (8)
N+	173 (50)	51 (15)	30 (9)
**Disease stage**			
I-II	23 (7)	4 (1)	11 (3)
III-IV	206 (59)	56 (16)	48 (14)
**Histological grade**			
Well	104 (30)	12 (3)	19 (6)
Moderate	85 (25)	26 (8)	28 (8)
Poor	39 (11)	22 (6)	12 (3)
**Follow-up**			
Mean	34	29	41
Median	19	19	37
Range	1 – 216	1 – 95	1 – 97
**Postoperative radiotherapy**			
No	75 (22)	16 (4)	41 (12)
Yes	154 (44)	44 (13)	18 (5)

Supplementary Table S2. Primers used for real-time RT-PCR.

Gene	Seq (5' → 3')
**NANOG1-Fw**	TGAGCTGGTTGCCTCATGTTAT
**NANOG1-Rv**	GAAGGAAAAGTATCAAGAAATTGGGATA
**SOX2-Fw**	TCAGGAGTTGTCAAGGCAGAGAAG
**SOX2-Rv**	CTCAGTCCTAGTCTTAAAGAGGCAGC
**RPL19-Fw**	GCGGAAGGGTACAGCCAAT
**RPL19-Rv**	GCAGCCGGCGCAAA

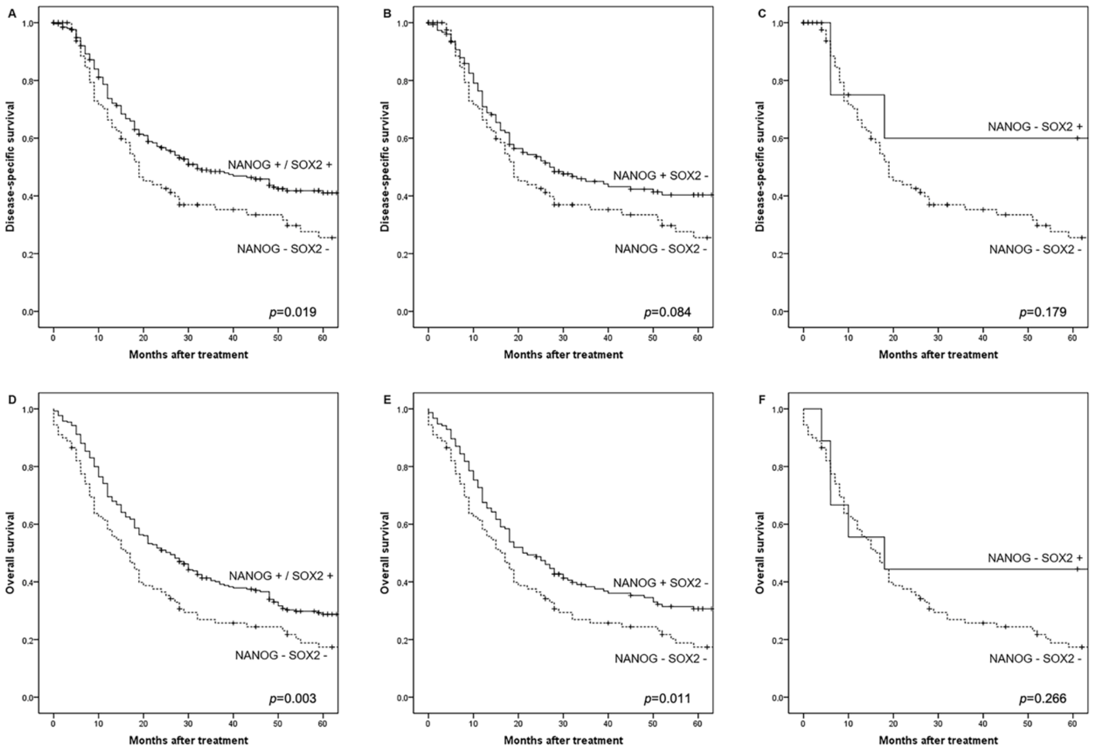

Supplementary Figure S1. Kaplan-Meier survival curves based on independent and combined expression of NANOG and SOX2. 5-year disease-specific (**A-C**) and overall (**D-F**) survival curves comparing double negative cases (NANOG-negative/SOX2-negative) with double positive cases (NANOG-positive/SOX2-positive) (**A, D**), exclusively NANOG-positive expression (NANOG-positive/SOX2-negative) (**B, E**) or SOX2-positive expression (NANOG-negative/SOX2-positive) **(C, F)**.

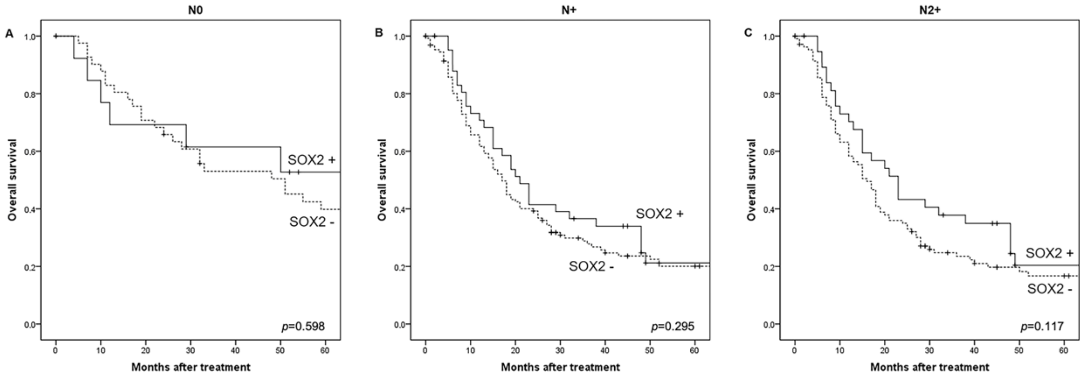

Supplementary Figure S2. Kaplan-Meier overall survival curves categorized by SOX2 expression in the subgroups N0 (**A**), N+ (**B**) and N2+ (**C**).

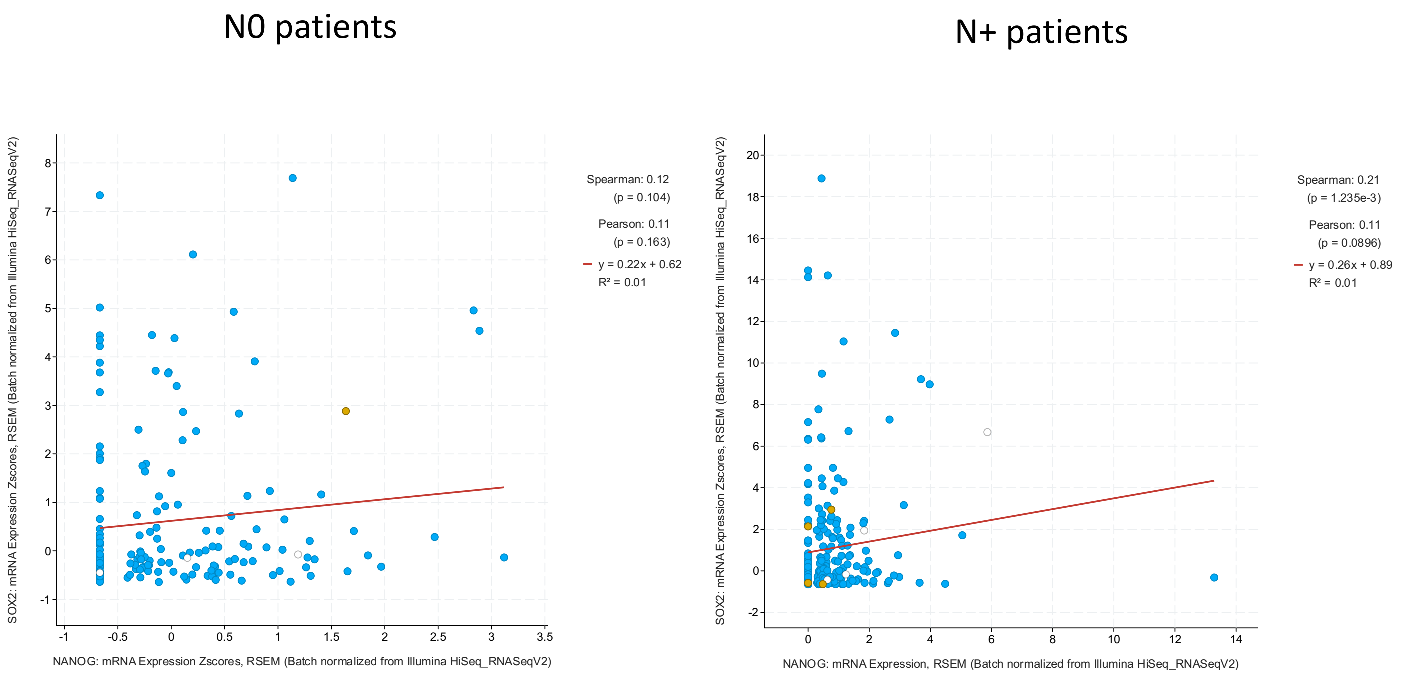

Supplementary Figure S3. Correlation between NANOG and SOX2 mRNA expression in a cohort of 530 HNSCC patients from The Cancer Genome Atlas (TCGA) [24] using the platform cBioPortal [25]. Scatter graphs and correlation between NANOG and SOX2 mRNA expression (RSEM RNAseqV2) in the subgroups of N0 and N+ patients are shown.

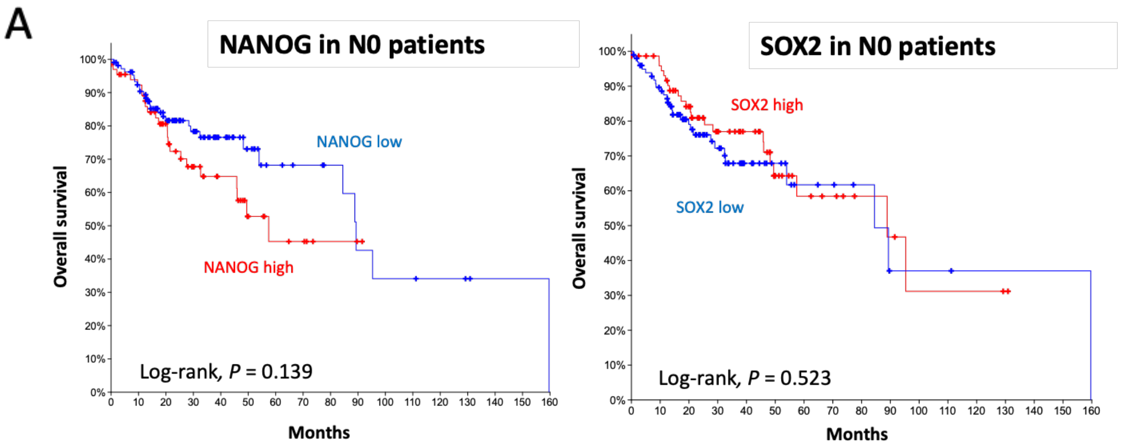

Patient Groups	Total Cases	Deceased Cases	Median Survival
High NANOG	**67**	**24**	**57.47**
Low NANOG	**110**	**28**	**89.32**

Patient Groups	Total Cases	Deceased Cases	Median Survival
High SOX2	**74**	**22**	**88.86**
Low SOX2	**103**	**30**	**84.49**

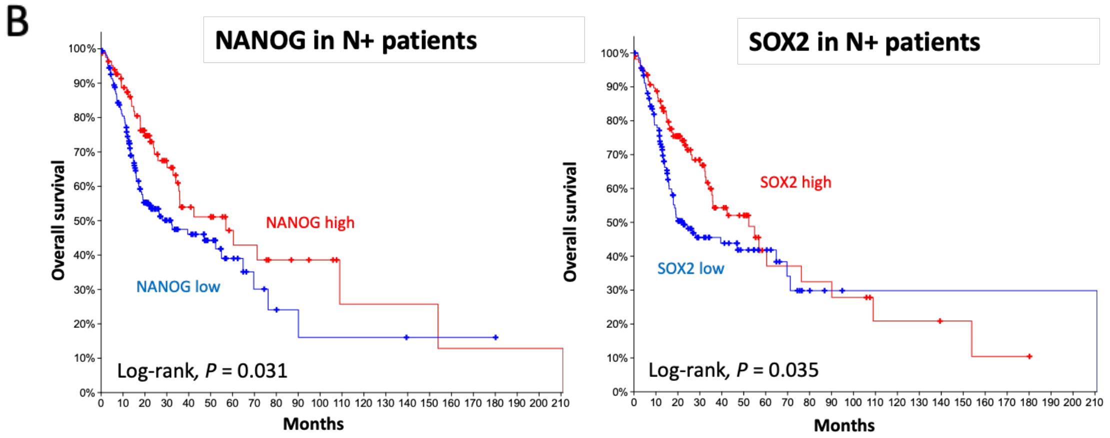

Patient Groups	Total Cases	Deceased Cases	Median Survival
High NANOG	**82**	**36**	**56.94**
Low NANOG	**164**	**81**	**32.21**

Patient Groups	Total Cases	Deceased Cases	Median Survival
High SOX2	**109**	**47**	**52.30**
Low SOX2	**137**	**70**	**22.35**

Supplementary Figure S4. Prognostic implications of NANOG and SOX2 mRNA expression in a subset of 424 HNSCC patients with lymph node stage data from the TCGA HNSCC cohort [24]. (**A**) Kaplan–Meier survival curves categorized by NANOG and SOX2 mRNA expression (RSEM RNAseqV2) dichotomized as high mRNA levels (above the median) versus low mRNA levels (below the median) in N0 patients (n = 177). (**B**) Kaplan–Meier survival curves categorized by NANOG and SOX2 mRNA expression (RSEM RNAseqV2) dichotomized as high mRNA levels (above the median) versus low mRNA levels (below the median) in N+ patients (n = 246).

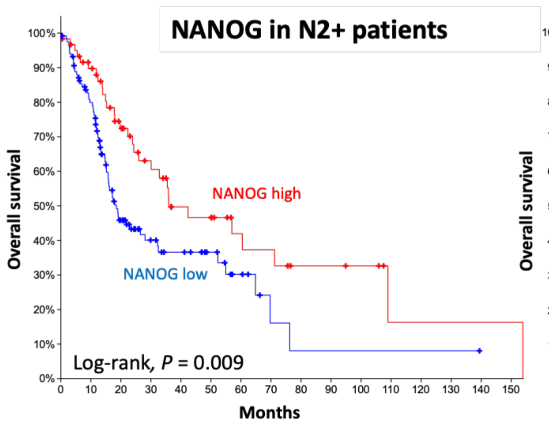

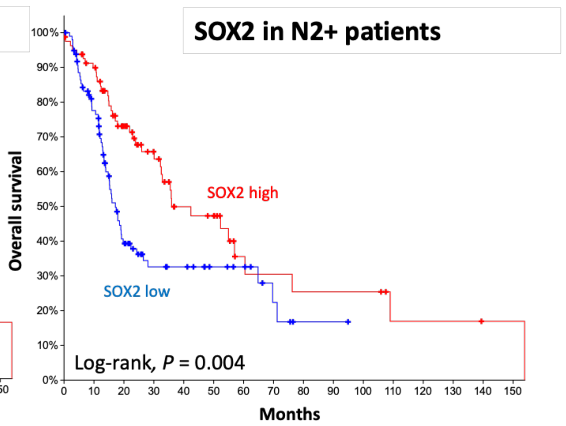

Patient Groups	Total Cases	Deceased Cases	Median Survival
High NANOG	**60**	**30**	**35.93**
Low NANOG	**119**	**68**	**18.54**

Patient Groups	Total Cases	Deceased Cases	Median Survival
High SOX2	**56**	**29**	**35.47**
Low SOX2	**123**	**69**	**19.19**

Supplementary Figure S5. Prognostic implications of NANOG and SOX2 mRNA expression in the subset of 179 N2+ patients from the TCGA HNSCC cohort [24]. Kaplan–Meier survival curves categorized by NANOG and SOX2 mRNA expression (RSEM RNAseqV2) dichotomized as high mRNA levels (above the median) versus low mRNA levels (below the median) in N2+ patients (n = 179).

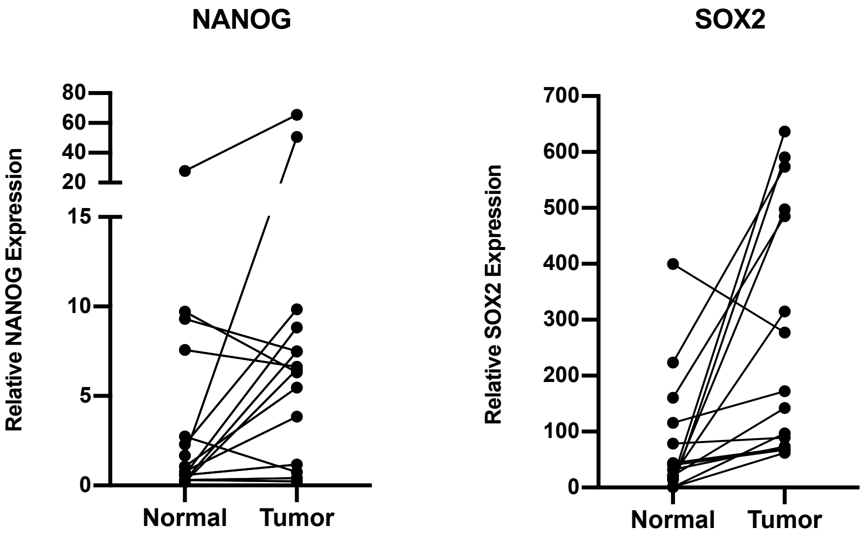

Supplementary Figure S6. Analysis of NANOG and SOX2 mRNA levels by real-time RT-PCR in 15 fresh HNSCC tumor samples and patient-matched normal epithelia. NANOG and SOX2 mRNA levels were normalized to L19 levels and data represented as fold-change relative to the primary keratinocytes, which was used as non-oncologic control and assigned with a value of 1.
